# Bis[5-methyl-2,3-bis­(thio­phen-2-yl)quinoxaline-κ*N* ^1^](nitrato-κ^2^ *O*,*O*′)silver(I)

**DOI:** 10.1107/S2414314623002651

**Published:** 2023-03-23

**Authors:** Guy Crundwell, Kristin M. Ellis

**Affiliations:** a Department of Chemistry & Biochemistry, Central Connecticut State University, 1619 Stanley Street, New Britain, CT 06053, USA; Goethe-Universität Frankfurt, Germany

**Keywords:** crystal structure, thienylquinoxaline, silver nitrate

## Abstract

The crystal structure of the title silver(I) complex, [Ag(NO_3_)(C_17_H_12_N_2_S_2_)_2_], has monoclinic (*C*2/*c*) symmetry, with the silver(I) atom and the nitrate group sitting on a twofold rotation axis. The complex also exhibits a thienyl-ring flip disorder, which is common for unsubstituted thio­phene rings.

## Abstract 2

In the title compound, the silver(I) metal center sits on a twofold rotation axis, as does the coordinated nitrato anion. The two 2-thienyl groups of the ligand make dihedral angles of 17.14 (9) and 77.55 (6)° with respect to the quinoxaline plane. The thienyl group that is less planar with the quinoxaline ring exhibits ring-flip disorder.

## Graphical abstract

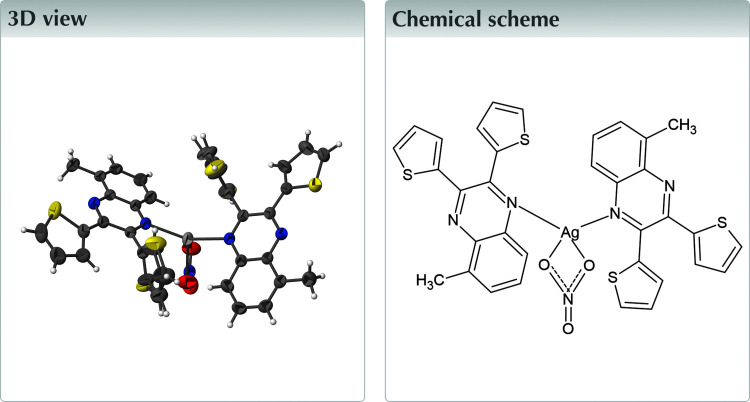

## Structure description

**Figure 1:**
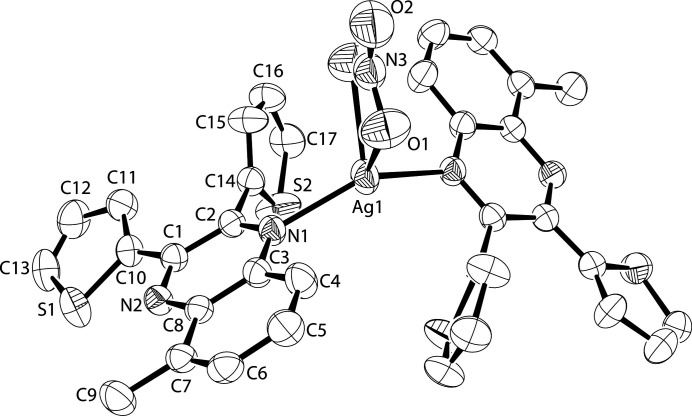
The molecular structure of the title compound. Displacement ellipsoids are drawn at the 50% probability level. Only symmetry-independent atoms are labeled. H atoms and the minor occupied sites of the disordered thienyl ring are omitted.

The central silver(I) atom and the nitrate anion sit on a twofold rotation axis. The two thienyl rings make dihedral angles of 17.14 (9) and 77.55 (6)° with respect to the quinoxaline moiety. The latter thienyl ring also has a flip disorder of 64.7 (4)%, which is common for unsubstituted thienyl rings (Crundwell *et al.*, 2003 ▸). The nitrate anion bonds to the silver *via* two O atoms. As seen with similar bis-dithienylquinoxaline silver nitrate complexes (Crundwell *et al.*, 2014 ▸), the N—Ag—N angle is correlated to the nitrate anion bonding to the metal in a bidentate fashion (Table 1 ▸, Fig. 1 ▸).

## Synthesis and crystallization

Crystals were grown by combining warmed methano­lic solutions of AgNO_3_ and 5-methyl-2,3-(di­thio­phen-2-yl)-quinoxaline in a 1:2 molar ratio. The combined solution was pipetted into test tubes, which were then placed into amber vials and loosely sealed until small colorless crystals were observed. Crystals were harvested and used immediately since the silver salts deteriorate in light within days.

## Refinement

Crystal data, data collection and structure refinement details are summarized in Table 2 ▸. Positional restraints and displace­ment parameter constraints were used in order to refine the amount of flip disorder, which was 64.7 (4)% for one of the thienyl rings (C14–C17, S2).

## Supplementary Material

Crystal structure: contains datablock(s) I. DOI: 10.1107/S2414314623002651/bt4136sup1.cif

Structure factors: contains datablock(s) I. DOI: 10.1107/S2414314623002651/bt4136Isup2.hkl

CCDC reference: 2250090

Additional supporting information:  crystallographic information; 3D view; checkCIF report

## Figures and Tables

**Table 1:** Selected geometric parameters (Å, °)

Ag1—O1	2.533 (2)	Ag1—N1	2.2619 (17)
Ag1—O1^i^	2.533 (2)	O1—N3	1.232 (3)
Ag1—N1^i^	2.2619 (17)	O2—N3	1.224 (4)
			
O1^i^—Ag1—O1	49.30 (9)	N1^i^—Ag1—O1^i^	103.14 (7)
N1^i^—Ag1—O1	109.40 (7)	N1—Ag1—O1	103.14 (7)
N1—Ag1—O1^i^	109.41 (7)	N1—Ag1—N1^i^	144.14 (9)

Symmetry code: (i) 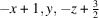 .

**Table 2:** Experimental details

Crystal data	
Chemical formula	[Ag(NO_3_)(C_17_H_12_N_2_S_2_)_2_]
*M*_r_	786.69
Crystal system, space group	Monoclinic, *C*2/*c*
Temperature (K)	293
*a*, *b*, *c* (Å)	18.9246 (10), 8.9789 (4), 22.2776 (13)
β (°)	120.967 (7)
*V* (Å^3^)	3245.9 (3)
*Z*	4
Radiation type	Mo *K*α
μ (mm^−1^)	0.92
Crystal size (mm)	0.38 × 0.35 × 0.20

Data collection	
Diffractometer	Xcalibur, Sapphire3
Absorption correction	Multi-scan (*CrysAlis PRO*; Oxford Diffraction, 2009 ▸)
*T*_min_, *T*_max_	0.886, 1.000
No. of measured, independent and observed [*I* > 2σ(*I*)] reflections	11880, 5743, 3896
*R*_int_	0.024
(sin θ/λ)_max_ (Å^−1^)	0.777

Refinement	
*R*[*F* ^2^ > 2σ(*F* ^2^)], *wR*(*F* ^2^), *S*	0.041, 0.107, 1.04
No. of reflections	5743
No. of parameters	228
No. of restraints	30
H-atom treatment	H-atom parameters constrained
Δρ_max_, Δρ_min_ (e Å^−3^)	0.32, −0.46

Computer programs: *CrysAlis CCD* (Oxford Diffraction, 2009 ▸), *CrysAlis RED* (Oxford Diffraction, 2009 ▸), *SHELXS97* (Sheldrick, 2008 ▸), *SHELXL* (Sheldrick, 2015 ▸), *OLEX2* (Dolomanov *et al.*, 2009 ▸), and *ORTEP-3 for Windows* (Farrugia, 2012 ▸).

## full crystallographic data

### Crystal data

**Table:**

[Ag(NO_3_)(C_17_H_12_N_2_S_2_)_2_]	*F*(000) = 1592
*M**_r_* = 786.69	*D*_x_ = 1.610 Mg m^−^^3^
Monoclinic, *C*2/*c*	Mo *K*α radiation, λ = 0.71073 Å
*a* = 18.9246 (10) Å	Cell parameters from 3173 reflections
*b* = 8.9789 (4) Å	θ = 4.5–31.7°
*c* = 22.2776 (13) Å	µ = 0.92 mm^−^^1^
β = 120.967 (7)°	*T* = 293 K
*V* = 3245.9 (3) Å^3^	Block, colorless
*Z* = 4	0.38 × 0.35 × 0.20 mm

### Data collection

**Table:**

Xcalibur, Sapphire3 diffractometer	5743 independent reflections
Radiation source: fine-focus sealed X-ray tube, Enhance (Mo) X-ray Source	3896 reflections with *I* > 2σ(*I*)
Graphite monochromator	*R*_int_ = 0.024
Detector resolution: 16.1790 pixels mm^-1^	θ_max_ = 33.5°, θ_min_ = 4.1°
ω scans	*h* = −25→28
Absorption correction: multi-scan (CrysAlisPro; Oxford Diffraction, 2009)	*k* = −13→9
*T*_min_ = 0.886, *T*_max_ = 1.000	*l* = −34→19
11880 measured reflections	

### Refinement

**Table:**

Refinement on *F*^2^	Primary atom site location: structure-invariant direct methods
Least-squares matrix: full	Secondary atom site location: difference Fourier map
*R*[*F*^2^ > 2σ(*F*^2^)] = 0.041	Hydrogen site location: inferred from neighbouring sites
*wR*(*F*^2^) = 0.107	H-atom parameters constrained
*S* = 1.04	*w* = 1/[σ^2^(*F*_o_^2^) + (0.044*P*)^2^ + 0.9845*P*] where *P* = (*F*_o_^2^ + 2*F*_c_^2^)/3
5743 reflections	(Δ/σ)_max_ < 0.001
228 parameters	Δρ_max_ = 0.32 e Å^−^^3^
30 restraints	Δρ_min_ = −0.46 e Å^−^^3^

### Special details

**Table:**

Experimental. Hydrogen atoms on sp^2^ carbons were included in calculated positions with a C-H distance of 0.93 Å and were included in the refinement in riding motion approximation with U_iso_ = 1.2U_eq_ of the carrier atom and hydrogen atoms on sp^3^ carbons were included in calculated positions with a C-H distance of 0.96 Å and were included in the refinement in riding motion approximation with U_iso_ = 1.5U_eq_ of the carrier atom.
Geometry. All esds (except the esd in the dihedral angle between two l.s. planes) are estimated using the full covariance matrix. The cell esds are taken into account individually in the estimation of esds in distances, angles and torsion angles; correlations between esds in cell parameters are only used when they are defined by crystal symmetry. An approximate (isotropic) treatment of cell esds is used for estimating esds involving l.s. planes.
Refinement. Refinement of F^2^ against ALL reflections. The weighted R-factor wR and goodness of fit S are based on F^2^, conventional R-factors R are based on F, with F set to zero for negative F^2^. The threshold expression of F^2^ > 2sigma(F^2^) is used only for calculating R-factors(gt) etc. and is not relevant to the choice of reflections for refinement. R-factors based on F^2^ are statistically about twice as large as those based on F, and R- factors based on ALL data will be even larger.

### Fractional atomic coordinates and isotropic or equivalent isotropic displacement parameters (Å^2^)

**Table:**

	*x*	*y*	*z*	*U*_iso_*/*U*_eq_	Occ. (<1)
Ag1	0.5000	0.28104 (3)	0.7500	0.05147 (10)	
O1	0.47816 (15)	0.5374 (2)	0.78512 (12)	0.0785 (6)	
O2	0.5000	0.7444 (4)	0.7500	0.0920 (11)	
N1	0.37028 (10)	0.2035 (2)	0.67321 (9)	0.0397 (4)	
N2	0.21614 (10)	0.0848 (2)	0.57801 (9)	0.0404 (4)	
N3	0.5000	0.6080 (4)	0.7500	0.0537 (7)	
C1	0.27757 (12)	0.0778 (2)	0.56570 (10)	0.0386 (4)	
C2	0.35742 (12)	0.1362 (2)	0.61561 (10)	0.0378 (4)	
C3	0.30625 (13)	0.2174 (2)	0.68498 (11)	0.0396 (4)	
C4	0.31805 (15)	0.2932 (3)	0.74494 (12)	0.0517 (6)	
H4	0.3686	0.3366	0.7761	0.062*	
C5	0.25366 (16)	0.3015 (3)	0.75613 (13)	0.0531 (6)	
H5	0.2608	0.3511	0.7955	0.064*	
C6	0.17693 (15)	0.2370 (3)	0.70951 (13)	0.0467 (5)	
H6	0.1348	0.2435	0.7192	0.056*	
C7	0.16241 (12)	0.1645 (2)	0.64991 (11)	0.0406 (4)	
C8	0.22894 (12)	0.1547 (2)	0.63735 (10)	0.0367 (4)	
C9	0.08016 (14)	0.0988 (3)	0.59912 (13)	0.0571 (6)	
H9A	0.0870	−0.0036	0.5906	0.086*	
H9B	0.0558	0.1532	0.5559	0.086*	
H9C	0.0449	0.1045	0.6182	0.086*	
C10	0.25759 (13)	0.0051 (3)	0.49975 (11)	0.0431 (5)	
C11	0.29678 (16)	0.0086 (3)	0.46086 (12)	0.0536 (6)	
H11	0.3452	0.0600	0.4739	0.064*	
C12	0.25216 (19)	−0.0774 (3)	0.39869 (14)	0.0667 (7)	
H12	0.2688	−0.0887	0.3663	0.080*	
C13	0.18427 (16)	−0.1403 (3)	0.39128 (13)	0.0619 (7)	
H13	0.1490	−0.2000	0.3534	0.074*	
S1	0.16912 (4)	−0.10019 (8)	0.45797 (3)	0.06093 (18)	
C14	0.42926 (12)	0.1231 (2)	0.60653 (11)	0.0405 (4)	0.647 (4)
C15	0.4616 (7)	0.2282 (9)	0.5845 (7)	0.0562 (8)	0.647 (4)
H15	0.4423	0.3255	0.5739	0.067*	0.647 (4)
C16	0.529 (2)	0.173 (2)	0.579 (3)	0.0586 (11)	0.647 (4)
H16	0.5563	0.2273	0.5614	0.070*	0.647 (4)
C17	0.5493 (8)	0.0331 (15)	0.6030 (10)	0.061 (2)	0.647 (4)
H17	0.5946	−0.0181	0.6076	0.073*	0.647 (4)
S2	0.48188 (14)	−0.03878 (19)	0.62341 (13)	0.0742 (5)	0.647 (4)
C14B	0.42926 (12)	0.1231 (2)	0.60653 (11)	0.0405 (4)	0.353 (4)
C15B	0.4753 (11)	−0.0013 (15)	0.6156 (11)	0.0742 (5)	0.353 (4)
H15B	0.4699	−0.0908	0.6339	0.089*	0.353 (4)
C16B	0.5332 (17)	0.024 (3)	0.593 (2)	0.061 (2)	0.353 (4)
H16B	0.5654	−0.0503	0.5902	0.073*	0.353 (4)
C17B	0.536 (4)	0.167 (4)	0.577 (5)	0.0586 (11)	0.353 (4)
H17B	0.5746	0.2062	0.5680	0.070*	0.353 (4)
S2B	0.4599 (3)	0.2654 (4)	0.5764 (3)	0.0562 (8)	0.353 (4)

### Atomic displacement parameters (Å^2^)

**Table:**

	*U*^11^	*U*^22^	*U*^33^	*U*^12^	*U*^13^	*U*^23^
Ag1	0.03173 (12)	0.0712 (2)	0.04456 (14)	0.000	0.01473 (10)	0.000
O1	0.0970 (16)	0.0848 (14)	0.0859 (15)	0.0033 (12)	0.0700 (14)	0.0022 (11)
O2	0.093 (3)	0.061 (2)	0.092 (2)	0.000	0.026 (2)	0.000
N1	0.0327 (8)	0.0489 (10)	0.0362 (8)	−0.0001 (7)	0.0168 (7)	−0.0003 (7)
N2	0.0359 (8)	0.0433 (10)	0.0414 (9)	−0.0012 (7)	0.0195 (7)	−0.0022 (7)
N3	0.0424 (14)	0.065 (2)	0.0481 (15)	0.000	0.0195 (12)	0.000
C1	0.0357 (10)	0.0393 (11)	0.0386 (10)	0.0009 (8)	0.0175 (8)	0.0012 (8)
C2	0.0332 (9)	0.0411 (11)	0.0384 (10)	0.0015 (8)	0.0180 (8)	0.0027 (8)
C3	0.0347 (9)	0.0469 (11)	0.0375 (9)	−0.0015 (9)	0.0188 (8)	−0.0006 (9)
C4	0.0449 (12)	0.0678 (16)	0.0446 (11)	−0.0105 (11)	0.0246 (10)	−0.0127 (11)
C5	0.0558 (14)	0.0644 (16)	0.0489 (12)	−0.0041 (12)	0.0340 (11)	−0.0090 (11)
C6	0.0461 (12)	0.0529 (13)	0.0517 (12)	0.0041 (10)	0.0328 (11)	0.0064 (10)
C7	0.0357 (10)	0.0422 (11)	0.0454 (11)	0.0016 (8)	0.0219 (9)	0.0064 (9)
C8	0.0345 (9)	0.0369 (10)	0.0382 (9)	0.0015 (8)	0.0184 (8)	0.0040 (8)
C9	0.0414 (12)	0.0742 (17)	0.0593 (14)	−0.0112 (11)	0.0284 (11)	−0.0076 (13)
C10	0.0390 (10)	0.0451 (12)	0.0404 (10)	0.0034 (9)	0.0169 (9)	−0.0024 (8)
C11	0.0591 (14)	0.0626 (15)	0.0425 (12)	−0.0111 (12)	0.0287 (11)	−0.0110 (11)
C12	0.0739 (19)	0.0817 (19)	0.0456 (13)	0.0012 (15)	0.0314 (13)	−0.0095 (13)
C13	0.0509 (14)	0.0680 (17)	0.0461 (12)	0.0061 (13)	0.0102 (11)	−0.0152 (12)
S1	0.0405 (3)	0.0738 (4)	0.0590 (4)	−0.0038 (3)	0.0189 (3)	−0.0197 (3)
C14	0.0351 (10)	0.0472 (12)	0.0405 (10)	0.0025 (8)	0.0203 (8)	0.0002 (9)
C15	0.0572 (9)	0.043 (2)	0.0860 (17)	0.0055 (13)	0.0495 (10)	0.0135 (13)
C16	0.053 (5)	0.066 (2)	0.073 (3)	−0.0032 (16)	0.043 (3)	0.003 (3)
C17	0.043 (5)	0.068 (2)	0.081 (6)	0.014 (3)	0.038 (6)	0.005 (3)
S2	0.0757 (8)	0.0617 (11)	0.1139 (11)	0.0299 (7)	0.0693 (9)	0.0374 (8)
C14B	0.0351 (10)	0.0472 (12)	0.0405 (10)	0.0025 (8)	0.0203 (8)	0.0002 (9)
C15B	0.0757 (8)	0.0617 (11)	0.1139 (11)	0.0299 (7)	0.0693 (9)	0.0374 (8)
C16B	0.043 (5)	0.068 (2)	0.081 (6)	0.014 (3)	0.038 (6)	0.005 (3)
C17B	0.053 (5)	0.066 (2)	0.073 (3)	−0.0032 (16)	0.043 (3)	0.003 (3)
S2B	0.0572 (9)	0.043 (2)	0.0860 (17)	0.0055 (13)	0.0495 (10)	0.0135 (13)

### Geometric parameters (Å, º)

**Table:**

Ag1—O1	2.533 (2)	C9—H9B	0.9600
Ag1—O1^i^	2.533 (2)	C9—H9C	0.9600
Ag1—N1^i^	2.2619 (17)	C10—C11	1.401 (3)
Ag1—N1	2.2619 (17)	C10—S1	1.720 (2)
O1—N3	1.232 (3)	C11—H11	0.9300
O2—N3	1.224 (4)	C11—C12	1.423 (3)
N1—C2	1.323 (3)	C12—H12	0.9300
N1—C3	1.371 (3)	C12—C13	1.334 (4)
N2—C1	1.326 (3)	C13—H13	0.9300
N2—C8	1.367 (3)	C13—S1	1.690 (3)
N3—O1^i^	1.232 (3)	C14—C15	1.348 (8)
C1—C2	1.437 (3)	C14—S2	1.692 (2)
C1—C10	1.468 (3)	C15—H15	0.9300
C2—C14	1.479 (3)	C15—C16	1.437 (11)
C3—C4	1.411 (3)	C16—H16	0.9300
C3—C8	1.409 (3)	C16—C17	1.337 (9)
C4—H4	0.9300	C17—H17	0.9300
C4—C5	1.366 (3)	C17—S2	1.688 (10)
C5—H5	0.9300	C15B—H15B	0.9300
C5—C6	1.404 (4)	C15B—C16B	1.437 (16)
C6—H6	0.9300	C16B—H16B	0.9300
C6—C7	1.373 (3)	C16B—C17B	1.335 (14)
C7—C8	1.426 (3)	C17B—H17B	0.9300
C7—C9	1.496 (3)	C17B—S2B	1.679 (16)
C9—H9A	0.9600		
			
O1^i^—Ag1—O1	49.30 (9)	C7—C9—H9B	109.5
N1^i^—Ag1—O1	109.40 (7)	C7—C9—H9C	109.5
N1—Ag1—O1^i^	109.41 (7)	H9A—C9—H9B	109.5
N1^i^—Ag1—O1^i^	103.14 (7)	H9A—C9—H9C	109.5
N1—Ag1—O1	103.14 (7)	H9B—C9—H9C	109.5
N1—Ag1—N1^i^	144.14 (9)	C1—C10—S1	117.63 (16)
N3—O1—Ag1	96.32 (18)	C11—C10—C1	131.5 (2)
C2—N1—Ag1	117.43 (13)	C11—C10—S1	110.81 (16)
C2—N1—C3	119.18 (17)	C10—C11—H11	124.7
C3—N1—Ag1	123.33 (13)	C10—C11—C12	110.6 (2)
C1—N2—C8	118.92 (17)	C12—C11—H11	124.7
O1^i^—N3—O1	118.1 (3)	C11—C12—H12	123.2
O2—N3—O1^i^	120.97 (17)	C13—C12—C11	113.7 (2)
O2—N3—O1	120.97 (17)	C13—C12—H12	123.2
N2—C1—C2	120.49 (18)	C12—C13—H13	123.5
N2—C1—C10	115.22 (18)	C12—C13—S1	112.9 (2)
C2—C1—C10	124.28 (18)	S1—C13—H13	123.5
N1—C2—C1	120.83 (18)	C13—S1—C10	91.99 (13)
N1—C2—C14	116.47 (17)	C2—C14—S2	121.03 (17)
C1—C2—C14	122.69 (18)	C15—C14—C2	128.1 (5)
N1—C3—C4	120.00 (19)	C15—C14—S2	110.9 (4)
N1—C3—C8	119.80 (18)	C14—C15—H15	123.8
C8—C3—C4	120.20 (19)	C14—C15—C16	112.3 (8)
C3—C4—H4	120.7	C16—C15—H15	123.8
C5—C4—C3	118.5 (2)	C15—C16—H16	124.0
C5—C4—H4	120.7	C17—C16—C15	112.0 (10)
C4—C5—H5	119.2	C17—C16—H16	124.0
C4—C5—C6	121.5 (2)	C16—C17—H17	124.2
C6—C5—H5	119.2	C16—C17—S2	111.6 (8)
C5—C6—H6	119.1	S2—C17—H17	124.2
C7—C6—C5	121.8 (2)	C17—S2—C14	93.0 (4)
C7—C6—H6	119.1	C16B—C15B—H15B	124.4
C6—C7—C8	117.46 (19)	C15B—C16B—H16B	123.7
C6—C7—C9	122.07 (19)	C17B—C16B—C15B	112.6 (18)
C8—C7—C9	120.47 (19)	C17B—C16B—H16B	123.7
N2—C8—C3	120.67 (17)	C16B—C17B—H17B	124.4
N2—C8—C7	118.87 (18)	C16B—C17B—S2B	111.1 (17)
C3—C8—C7	120.46 (18)	S2B—C17B—H17B	124.4
C7—C9—H9A	109.5		
			
Ag1—O1—N3—O1^i^	−0.002 (2)	C2—C1—C10—C11	−19.4 (4)
Ag1—O1—N3—O2	180.000 (2)	C2—C1—C10—S1	163.24 (17)
Ag1—N1—C2—C1	177.25 (14)	C2—C14—C15—C16	−177 (2)
Ag1—N1—C2—C14	−1.9 (2)	C2—C14—S2—C17	−179.4 (7)
Ag1—N1—C3—C4	5.0 (3)	C3—N1—C2—C1	−0.1 (3)
Ag1—N1—C3—C8	−174.50 (14)	C3—N1—C2—C14	−179.20 (18)
O1^i^—Ag1—O1—N3	0.001 (2)	C3—C4—C5—C6	−0.1 (4)
O1^i^—Ag1—N1—C2	85.90 (16)	C4—C3—C8—N2	178.0 (2)
O1—Ag1—N1—C2	137.02 (15)	C4—C3—C8—C7	−1.3 (3)
O1^i^—Ag1—N1—C3	−96.90 (16)	C4—C5—C6—C7	−1.0 (4)
O1—Ag1—N1—C3	−45.79 (17)	C5—C6—C7—C8	1.0 (3)
N1^i^—Ag1—O1—N3	90.85 (13)	C5—C6—C7—C9	−178.4 (2)
N1—Ag1—O1—N3	−104.43 (13)	C6—C7—C8—N2	−179.2 (2)
N1^i^—Ag1—N1—C2	−68.10 (14)	C6—C7—C8—C3	0.2 (3)
N1^i^—Ag1—N1—C3	109.09 (16)	C8—N2—C1—C2	3.0 (3)
N1—C2—C14—C15	−79.2 (8)	C8—N2—C1—C10	−178.15 (18)
N1—C2—C14—S2	101.6 (2)	C8—C3—C4—C5	1.3 (4)
N1—C3—C4—C5	−178.2 (2)	C9—C7—C8—N2	0.3 (3)
N1—C3—C8—N2	−2.5 (3)	C9—C7—C8—C3	179.6 (2)
N1—C3—C8—C7	178.19 (19)	C10—C1—C2—N1	178.41 (19)
N2—C1—C2—N1	−2.9 (3)	C10—C1—C2—C14	−2.5 (3)
N2—C1—C2—C14	176.19 (19)	C10—C11—C12—C13	0.3 (4)
N2—C1—C10—C11	161.8 (2)	C11—C10—S1—C13	0.7 (2)
N2—C1—C10—S1	−15.5 (3)	C11—C12—C13—S1	0.2 (3)
C1—N2—C8—C3	−0.4 (3)	C12—C13—S1—C10	−0.5 (2)
C1—N2—C8—C7	178.89 (19)	S1—C10—C11—C12	−0.6 (3)
C1—C2—C14—C15	101.7 (8)	C14—C15—C16—C17	−5 (4)
C1—C2—C14—S2	−77.6 (3)	C15—C14—S2—C17	1.2 (9)
C1—C10—C11—C12	−178.1 (2)	C15—C16—C17—S2	6 (4)
C1—C10—S1—C13	178.55 (18)	C16—C17—S2—C14	−4 (3)
C2—N1—C3—C4	−177.9 (2)	S2—C14—C15—C16	2 (2)
C2—N1—C3—C8	2.6 (3)	C15B—C16B—C17B—S2B	−9 (7)

Symmetry code: (i) −*x*+1, *y*, −*z*+3/2.
